# The prognostic role of systemic inflammatory markers on HIV-infected patients with non-Hodgkin lymphoma, a multicenter cohort study

**DOI:** 10.1186/s12967-015-0446-8

**Published:** 2015-03-14

**Authors:** Elena Raffetti, Francesco Donato, Filippo Castelnuovo, Nicoletta Ladisa, Giuseppe Paraninfo, Elisa Di Filippo, Daniela Segala, Giuliana Cologni, Alessandra Bandera, Fabio Zacchi, Simona Digiambenedetto, Massimo Di Pietro, Francesco Castelli, Eugenia Quiros-Roldan, MASTER Cohort

**Affiliations:** Unit of Hygiene, Epidemiology and Public Health, University of Brescia, Brescia, Italy; Hospital Division of Infectious and Tropical Diseases, Spedali Civili Hospital, Brescia, Italy; Department of Infectious Diseases, Polyclinic of Bari, University of Bari, Bari, Italy; University Division of Infectious and Tropical Diseases, University of Brescia, Brescia, Italy; Department of Infectious Diseases of Papa Giovanni XXIII Hospital, Bergamo, Italy; Department of Infectious Diseases, S. Anna Hospital, Ferrara, Italy; Department of Infectious Diseases, San Gerardo de’ Tintori” Hospital, Monza, Italy; Clinical Infectious Diseases, Istituti Ospitalieri, Cremona, Italy; Institute of Clinical Infectious Diseases, Polyclinic A. Gemelli, University of Sacred Heart, Rome, Italy; Department of Infectious Diseases, SM. Annunziata Hospital, Florence, Italy

**Keywords:** Inflammatory markers, Prognostic score, HIV, Non-Hodgkin lymphoma

## Abstract

**Background:**

The systemic inflammatory response has been postulated as having prognostic significance in a wide range of different cancer types. We aimed to assess the prognostic role of inflammatory markers on survival in HIV-infected patients with Non-Hodgkin Lymphoma (NHL), and to compute a prognostic score based on inflammatory biomarkers.

**Methods:**

We evaluated data on HIV patients with NLH diagnosis between 1998 and 2012 in a HIV Italian Cohort. Using Cox proportional regression model, we assessed the prognostic role of Neutrophil-Lymphocyte Ratio (NLR), Platelet-Lymphocyte Ratio (PLR), Glasgow Prognostic Score (GPS), modified Glasgow Prognostic Score (mGPS), Prognostic Index (PI), and Prognostic Nutritional Index (PNI). We also computed a risk score equation, assigning patients to a derivation and a validation sample. The area under the curve (AUC) was use to evaluate the predictive ability of this score.

**Results:**

215 non-Hodgkin lymphoma cases (80.0% males) with a mean age of 43.2 years were included. Deaths were observed in 98 (45.6%) patients during a median follow up of 5 years. GPS, mGPS, PI and PNI were independently associated with risk of death. We also computed a mortality risk score which included PNI and occurrence of an AIDS event within six months from NHL diagnosis. The AUCs were 0.69 (95% CI 0.58 to 0.81) and 0.69 (95% CI 0.57 to 0.81) at 3 and 5 years of the follow-up, respectively.

**Conclusions:**

GPS, mGPS, PI and PNI are independent prognostic factors for survival of HIV patients with NHL.

## Introduction

Non-Hodgkin Lymphoma (NHL) is one of the most common cancer and cause of death among HIV-infected patients [1-3]. Overall survival is poor and more than half subjects die within five years from NHL diagnosis, although it has improved after the introduction of the combined Antiretroviral Therapy (cART) [2-9].

The International Prognostic Index (IPI) was developed at the beginning of 1990s by the International Non-Hodgkin Lymphoma Prognostic Factors Project to create a prognostic tool for patients with aggressive NHL treated with doxorubicin-containing chemotherapy in the general population [10]. The IPI categorizes patients into low, intermediate and high risk groups based on the baseline characteristics including age, Eastern Cooperative Oncology Group performance status, Lactate Dehydrogenase (LDH) level, Ann Arbor stage, and extranodal involvement. The IPI has also been demonstrated prognostic for lymphomas associated with HIV infection [4].

Tumor microenvironment and inflammatory response have an important role at different stages of tumor development, including initiation, promotion, malignant conversion, invasion and metastasis [11]. Recent studies showed that some blood parameters associated with systemic inflammation, i.e. C-Reactive Protein (CRP), white cell, neutrophil and platelet count, and hypoalbuminemia are predictors of survival in patients with primary solid cancers [12]. Various combinations of these factors have been used to derive inflammation-based scores to predict survival of subjects with solid cancers [13-16], such as Glasgow Prognostic Score (GPS), Neutrophil/Lymphocyte Ratio (NLR) and Platelet/Lymphocyte Ratio (PLR).

Few studies have investigated the association between these inflammatory scores and survival in patients with NHL [17-19], none of which in HIV-infected patients, so far.

The aims of this study were: (i) to evaluate the prognostic role of inflammatory markers on survival in HIV-infected patients with NHL and, (ii) to compute a prognostic score based on demographical and clinical variables and inflammatory biomarkers.

## Methods

The Italian MASTER cohort is a hospital-based multicenter, open, dynamic cohort established in the mid-1990s with retrospective patients’ enrolment since 1986. At present, the cohort includes about 24 500 HIV-infected subjects, aged 18 years or older, at eight Infectious Diseases Units throughout Italy. In this work we included HIV-infected patients with a first NHL diagnosis between January 1998 and December 2012. The study design and cancer collection data have been described previously [1].

We retrieved gender, age, country of origin, HIV exposure risk, date of enrolment in the cohort, Hepatitis B Virus (HBV) or/and Hepatitis C Virus (HCV) co-infection and cART at cancer diagnosis from the MASTER electronic database. The following parameters, measured within 6 months from the diagnosis of NHL, were also retrieved: AIDS event occurrence, HIV-RNA, CD4 cell count, CD8 cell count, CRP, albumin, white blood cell, neutrophil, lymphocyte and platelet counts. The inflammatory based prognostic variables GPS, modified Glasgow Prognostic Score (mGPS), NLR, PLR, Prognostic Index (PI), and Prognostic Nutritional Index (PNI) were defined as shown in Table 1.

**Table 1:** **Inflammation-based prognostic variables**

**Variables**	**Criteria**	**Score**
**GPS**	C-Reactive Protein ≤1.0 mg/dl and albumin ≥3.5 g/dl	0
	C-Reactive Protein >1.0 mg/dl and albumin ≥3.5 g/dl	1
	C-Reactive Protein ≤1.0 mg/dl and albumin <3.5 g/dl	
	C-Reactive Protein >1.0 mg/dl and albumin <3.5 g/dl	2
**mGPS**	C-Reactive Protein ≤1.0 mg/dl	0
	C-Reactive Protein >1.0 mg/dl and albumin ≥3.5 g/dl	1
	C-Reactive Protein >1.0 mg/dl and albumin <3.5 g/dl	2
**NLR**	neutrophil coun/lymphocyte count <1/3	0
	neutrophil count/lymphocyte count 3-5/1	1
	neutrophil count/lymphocyte count ≥3/1	2
**PLR**	platelet count/lymphocyte count <150/1	0
	platelet count/lymphocyte count 150– 300/1	1
	platelet count/lymphocyte count >300/1	2
**PI**	C-Reactive Protein ≤1 mg/dl and white blood cell count ≤11,000/μl	0
	C-Reactive Protein ≤1 mg/dl and white blood cell count >11,000/μl	1
	C-Reactive Protein >1 mg/dl and white blood cell count ≤11,000/μl	
	C-Reactive Protein >1 mg/dl and white blood cell count >11,000/μl	2
**PNI**	albumin (g/dl) × 10 + 0.005 × total	0
	lymphocyte count (/μl) ≥45	
	albumin (g/dl) × 10 + 0.005 × total	1
	lymphocyte count (/μl) <45	

*Abbreviations*: *GPS* Glasgow Prognostic Score, *mGPS* modified Glasgow Prognostic Score, *NLR* Neutrophil/Lymphocyte Ratio, *PLR* Platelet/Lymphocyte Ratio, *PI* Prognostic Index, *PNI* Prognostic Nutritional Index.

The study was conducted in accordance with the guidelines of the Declaration of Helsinki and the principles of Good Clinical Practice. Informed consent was obtained according to the standards of the local ethics committees.

### Statistical analysis

The primary outcome of this study was all-cause mortality. The cumulative risk of death was determined from the data of NHL diagnosis to the end of the observation period. The censor date for survival analysis was 31st December 2012. The observation period ended either on 31st December 2012, or at last follow-up visit or death, whichever occurred first. Patients lost to follow-up contributed to the time at risk until last visit.

At first, the associations of each prognostic variable with all-cause mortality were tested by univariate and multivariate analysis using Cox proportional hazard models. Gender, age at cancer diagnosis, intravenous drug use, AIDS defining event, HBV and/or HCV co-infection, cART, CD4 cell count, HIV-RNA undetectable were included as covariates. The results were expressed as Hazards Ratios (HRs), their 95% Confidence Intervals (95% CIs), and p-values according to Wald test. Due a few missing data in GPS, mGPS, PLR, PI, and PNI, the Cox proportional hazard regression models were adjusted by stabilized inverse probability weights with missing data in prognostic variables considered missing at random [20]. In order to compare the fitness of the model with and without the inflammatory variables, we used the log-likelihood ratio test.

The Area Under the Receiver Operating Characteristic (ROC) Curve (AUC) was used to assess the predictive accuracy of each prognostic variable. As sensitivity analyses, we assessed the consistency of the prognostic role of inflammatory variables in subjects who underwent cART at NHL diagnosis.

Non-linear relationships between continuous prognostic variables and risk of death were assessed by Cox regression models with spline terms of the prognostic variables [21]. We used the Akaike’s information criterion [22] to assess fitting of models with linear and non-linear terms and to choose the number of spline knots.

Subsequently, we derived a risk score of mortality on the basis of the inflammatory, demographic and clinical variables. To this end, the patients were randomly divided into two subgroups by split-sample technique: (i) derivation subgroup including 70% of subjects, and (ii) validation subgroup, including 30% of subjects. In the derivation sample, a Cox proportional regression model was used to evaluate the association between baseline factors and risk of death, using a cut-off of minimal 10 events per variable for first selection of variables. Gender, age at cancer diagnosis, intravenous drug use, AIDS defining event, HBV and/or HCV co-infection, cART, CD4 cell count, HIV-RNA undetectable and PNI were included. Bootstrapping with 1000 replications was performed to check the stability of variables included in the final models. Linear prediction equations, representing the risk scores, were derived from the final model. For visualization purposes, three risk groups were created according to tertiles of the risk scores distribution in the derivation sample. Cumulative incidence rates of mortality among these three groups were compared by the Kaplan–Meier methods and tested with the log-rank test for trend in the derivation sample. In the derivation and the validation sample the AUC was used to assess the predictive accuracy of the risk scores.

The selection of variables for fitting the most parsimonious models was performed using a backward stepwise procedure, with p = 0.20 for retaining each variable in the model. A graphical check on each regressor did not detect major departures from the proportional hazard assumption of the model.

All the statistical tests were two-sided, assumed a level of significance of 0.05 and were performed using Stata version 12.0 (StataCorp, College Station, TX, USA).

## Results

A total of 215 non-Hodgkin lymphoma cases were included. They had a mean age of 43.2 years at time of NHL diagnosis (Standard Deviation, SD = 9.0) and most of them were males (80.0%). The characteristics of the patient population at NHL diagnosis are shown in Table 2. Most of them were assuming cART and plasmatic HIV-RNA was positive (>37 cp/ml). More than half patients had severe immunodeficiency with CD4 cell count less than <200 cells/mm3.

**Table 2:** **Demographical and clinical features at non-Hodgkin lymphoma diagnosis**

**Variables**	**Categories**	**n (%)**
**Total**		215
**Gender**	Male	172 (80.0)
**Age (years)**	<35	31 (14.4)
	35-49	136 (63.3)
	≥50	48 (22.3)
	Mean (SD)	43.2 (9.0)
**Period of Diagnosis**	1998-2002	71 (33.0)
	2003-2007	88 (40.9)
	2008-2012	56 (26.1)
**IDU**	Yes	96 (42.8)
**Migrants**	Yes	28 (8.7)
**Previous AIDS defining event**	Yes	81 (37.7)
**HBV or/and HCV co-infection**	Yes	111 (51.6)
**cART**	Yes	172 (80.0)
**CD4 cell count, cell/mm3**	<200	104 (54.2)
	≥200	88 (45.8)
	Mean (SD)	275.9 (235.3)
**CD4/CD8 Ratio**	<1	158 (89.3)
	≥1	19 (10.7)
	Mean (SD)	0.46 (0.50)
**HIV-RNA, copies/mL**	Undetectable	49 (26.1)
	Positive	139 (73.9)
	Mean (SD)*	113208.8 ( 310601.3)
**Status at the end of the follow-up**	Alive	117 (54.4)
	Dead	98 (45.6)

*Abbreviations*: *SD* Standard Deviation, *IDU* Intravenous Drug Use, *HBV* Hepatitis B Virus, *HCV* Hepatitis C Virus, *cART* combined Antiretroviral Therapy, *In subjects with positive HIV-RNA.

After a median follow up of 2 years (ranging from 2 days to 13.9 years), 18 patients (8.4%) were lost and 98 (45.6%) died. The median survival was 9.3 years. At 1, 3 and 5 years, cumulative risk of death was 34.1%, 44.3% and 45.7%, respectively.

### Prognostic role of inflammation-based prognostic variables

Table 3 describes the distribution of inflammatory markers and the association between each of them and risk of death using Cox regression models. Median time from NHL diagnosis to blood testing was 18 days (interquartile range: 3–53 days).

**Table 3:** **Univariate and multivariate Cox regression models for each inflammatory prognostic variable**

**Variables**	**Category** ^**$**^			**Univariate model**			**Multivariate model**	
		**n (%)** ^**#**^	**n**	**HR (95% CI)**	**P value**	**n**	**HR (95% CI)** ^**π**^	**P value**
GPS*	0	20 (25.3)	79	Ref		65	Ref	
	1	42 (53.2)		1.82 (0.49-6.69)	NS		1.91 (0.45-7.65)	NS
	2	17 (21.5)		4.39 (1.19-16.2)	0.026		4.78 (1.15-19.77)	0.031
	Available	79 (36.7)						
mGPS*	0	24 (30.4)	79	Ref		65	Ref	
	1	38 (48.1)		2.01 (0.61-7.00)	NS		2.07 (0.61-7.01)	NS
	2	17 (21.5)		4.46 (1.36-14.6)	0.013		4.35 (1.16-16.36)	0.029
	Available	79 (36.7)						
**NLR**	0	129 (67.2)	192	Ref				
	1	32 (16.7)		1.31 (0.74-2.30)	NS			
	2	31 (16.1)		1.48 (0.84-2.61)	NS			
	Mean (SD)	2.92 (2.79)						
	Available	192 (89.3)						
**PLR**	0	97 (48.3)	199	Ref				
	1	67 (33.3)		1.16 (0.71-1.90)	NS			
	2	37 (18.4)		1.58 (0.88-2.84)	NS			
	Mean (SD)	193.7 (135.1)						
	Available	199 (92.6)						
PI*	0	29 (31.9)	91	Ref		77	Ref	
	1	60 (65.9)		3.33 (1.12-9.93)	0.030		3.25 (1.06-9.94)	0.039
	2	2 (2.2)		3.98 (0.86-28.35)	NS		11.3 (1.03-124.9)	0.047
	Available	91 (42.3)						
PNI*	0	54 (39.7)	136	Ref		114	Ref	
	1	82 (60.3)		2.36 (1.23-4.53)	0.010		2.57 (1.25-5.30)	0.010
	Available	136 (63.3)						

*Abbreviations*: *HR* Hazard Ratio, *95% CI*, 95% Confidence Interval, *SD* Standard Deviation, *GPS* Glasgow Prognostic Score, *mGPS* modified Glasgow Prognostic Score, *NLR* Neutrophil/Lymphocyte Ratio, *PLR* Platelet/Lymphocyte Ratio, *PI* Prognostic Index, *PNI* Prognostic Nutritional Index, *NS* Not Statistical Significant (p > 0.05).

^#^Colum percentage**,** *Weighted Cox regression models, ^π^Model adjusted for gender, age at diagnosis, intravenous drug user, AIDS defining event, CD4 cell count and HIV-RNA undetectable. ^$^For prognostic variables classification see Table 1.

Score 0 was attributed to the majority of patients for NLR, while for PI and PNI most subjects were categorized as score 1. About half of patients were scored with 1 for GPS and mGPS, and with 0 for PLR. No associations were found between prognostic variables and HIV-RNA copy number in subjects with positive HIV-RNA at NHL diagnosis. GPS, mGPS, PI and PNI were independently associated with risk of death using both univariate and multivariate Cox models. These results were also confirmed when we limited the analyses to patients under cART (data not shown).

The likelihood ratio tests for the global fit of the model, before and after addition of inflammatory variables to the full model, were significant for GPS, mGPS, PI and PNI (p < 0.001).

**Figure 1:**
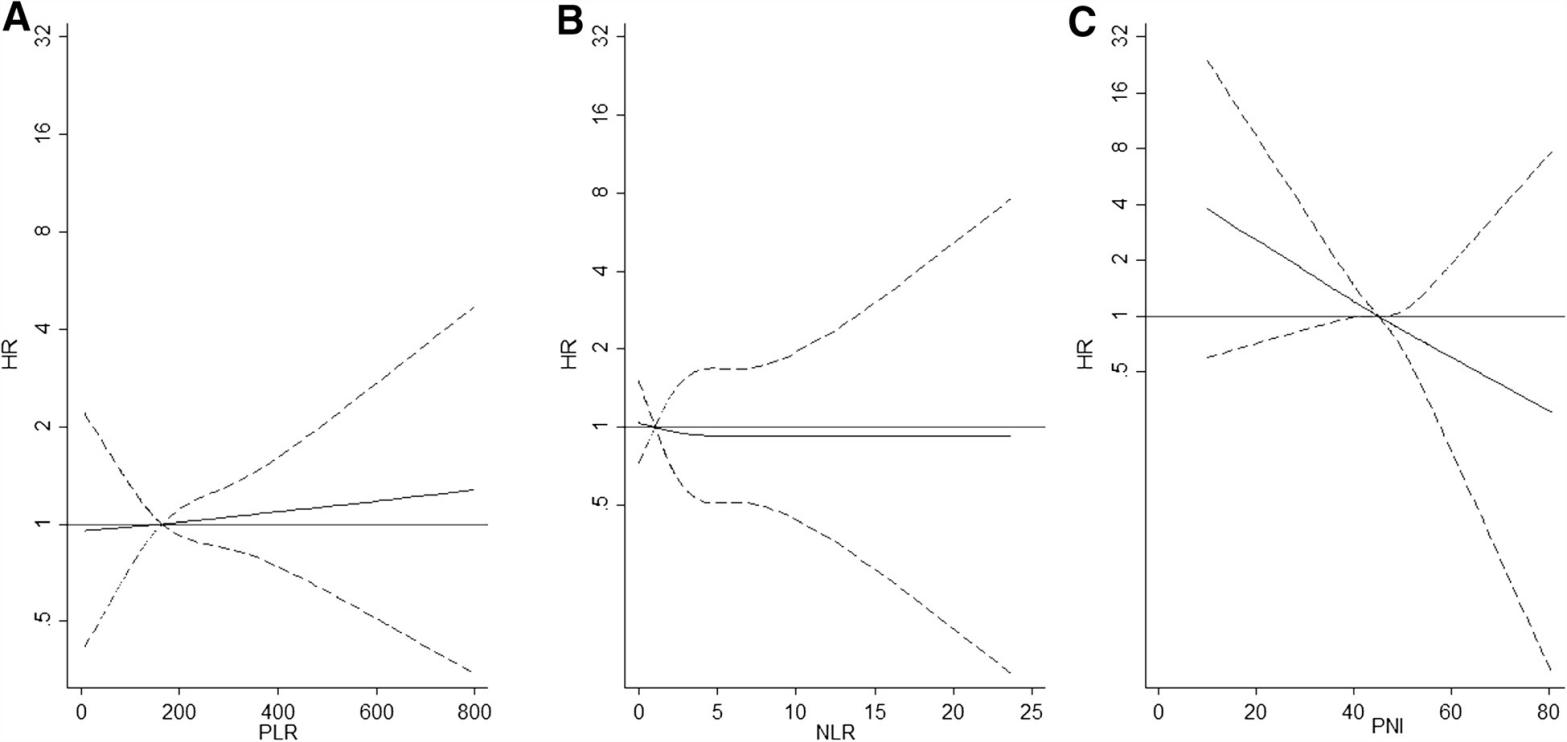
**The relationship between the inflammatory-based prognostic scores considered as continuous (PLR (B), NLR (A) and PNI (C)), and the hazard ratio of death (HR).** The HRs were computed in Cox regression models with cubic spline term for each prognostic score adjusted for gender, age at diagnosis, intravenous drug use, AIDS defining event, CD4 cell count, cART therapy prescription and HIV-RNA undetectable. The reference value for each spline term is 3 for NLR, 150 for PLR and 45 for PNI.

Non-linear relationships between NLR, PLR, PNI and risk of death were evaluated by using multivariate Cox regression models with a restricted cubic-spline for each prognostic score (Figure 1). A trend of increasing risk of death with increasing PNI was observed, although not statistical significant, whereas no trend with PLR and NLR was found.

The ROC analysis showed that mGPS, GPS, PNI and PI had statistically significant discrimination ability, with AUC ranging from 0.60 to 0.69, with follow-up truncated at both 3 and 5 years (Table 4).

**Table 4:** **Areas under the ROC curve using various prognostic variables**

**Prognostic variables**	**Area under the ROC curve at 3-year of follow-up ( 95% CI)**	**Area under the ROC curve at 5-years of follow-up ( 95% CI)**
GPS*	**0.67 (0.54-0.80)**	**0.67 (0.54-0.80)**
mGPS*	**0.69 (0.56-0.82)**	**0.66 (0.53-0.80)**
**NLR**	0.54 (0.46-0.62)	0.54 (0.47-0.62)
**PLR**	0.53 (0.45-0.60)	0.54 (0.477-0.62)
PI*	**0.63 (0.53-0.73)**	**0.60 (0.51-0.71)**
PNI*	**0.65 (0.55-0.74)**	**0.64 (0.55-0.74)**

*Abbreviations*: *ROC* Receiver Operating Characteristic, *95% CI* 95% Confidence Interval, *GPS* Glasgow Prognostic Score, *mGPS* modified Glasgow Prognostic Score, *NLR* Neutrophil/Lymphocyte Ratio, *PLR* Platelet/Lymphocyte Ratio, *PI* Prognostic Index, *PNI* Prognostic Nutritional Index.

The bold Areas under the ROC curves are those concerning variables resulted significantly predictive of death.

### Mortality risk score

Finally, we computed a risk score for death on the basis of the final Cox model. In the derivation sample (93 subjects with PNI available, 34 of which died), age, occurrence of an AIDS defining event within six months from date of NHL diagnosis, no cART assumption, CD4 cell count lower than 200 cells/mm3 and PNI equal to 1 were significantly associated with fatal outcome in univariate Cox regression analysis (Table 5). However, in multivariate analyses, only occurrence of AIDS defining event within 6 months from NHL diagnosis and PNI were independently associated with mortality, also using bootstrapping technique.

**Table 5:** **Cox regression analyses for mortality in the derivation sample**

**Variable**	**Category**	**Univariate analysis**		**Multivariate analysis** ^**π**^	
				**n subjects included = 93**	
		HR (95% CI)*	**P value**	HR (95% CI)*	**P value**
**Gender**	Male vs Female	1.03 (0.59-1.79)	NS		
**Age (years)**		1.02 (1.0-1.05)	NS		
**IDU**	Yes vs No	1.04 (0.66-1.66)	NS		
**AIDS defining event**	Yes vs No	1.73 (1.09-2.73)	0.019	2.02 (1.01-4.02)	0.045
**HBV or/and HCV co-infection**	Yes vs No	0.94 (0.60-1.49)	NS		
**cART**	Yes vs No	0.57 (0.35-0.95)	0.032		
**CD4 cell count, cell/mm3**	<200 vs ≥200	1.79 (1.12-2.88)	0.015		
**HIV-RNA undetectable**	Yes vs No	1.52 (0.84-2.73)	NS		
**PNI**	<45 vs ≥45	2.42 (1.22-4.77)	0.011	2.21 (1.11-4.40)	0.023

*Abbreviations*: *HR* Hazard Ratio, *95% CI* 95% Confidence Interval,* IDU* Intravenous Drug Use, *HBV* Hepatitis B Virus, *HCV* Hepatitis C Virus, *cART* combined Antiretroviral Therapy, *PNI* Prognostic Nutritional Index, *NS* Not Statistical Significant (p > 0.05).

*Adjusted for all the variables in the table. ^π^The mortality risk score based on the regression coefficients of the variables in final model was represented by 0.703*(AIDS defining event [yes =1/no = 0]) + 0.794 * (PNI) [AIDS defining event within six months from NHL diagnosis].

**Figure 2:**
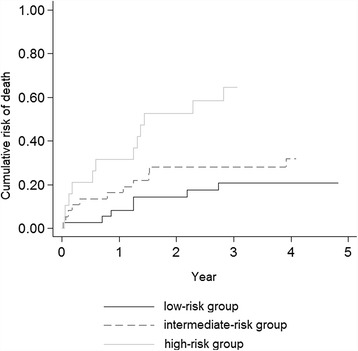
**Cumulative risk of death according to occurrence of AIDS defining event and PNI.** The low-risk group included patients without AIDS defining event within six months from NHL diagnosis and with PNI > 45; the intermediate-risk group included patients with AIDS defining event within six months from data of NHL diagnosis or with PNI < 45; and the high-risk group included patient with AIDS defining event within six months from NHL diagnosis and with PNI < 45.

The linear prediction equation for risk of mortality (Rm) derived from the final Cox model was: Rm = 0. 703 *(occurrence of AIDS defining event within six months from NHL diagnosis [yes =1/no = 0]) + 0.794* (PNI). The predictive accuracies of this risk score for mortality, as measured by the AUC, were 0.69 (95% CI 0.58 to 0.81) and 0.69 (0.57 to 0.81) in derivation sample, and 0.69 (0.49 to 0.90) and 0.73 (0.53 to 0.92) in validation sample (47 subjects with PNI available, 12 of which died), at 3 and 5 years of follow-up, respectively. As an application of the score, patients were categorized into three risk groups: patients without AIDS defining event within six months from the date of NHL diagnosis and with PNI > 45, patients with AIDS defining event within six months from the date of NHL diagnosis or with PNI < 45 and patients with AIDS defining event within six months from the date of NHL diagnosis and with PNI < 45. According to the 3 risk score categories, cumulative mortality rates were 20.8% (10.4 to 38.9), 31.8% (18.8 to 50.3) and 64.5% (43.3 to 84.8) in the low-, intermediate- and high-risk group (log rank test p < 0.001) (Figure 2).

As a sensitive analysis, we have also computed a prognostic score including PNI and AIDS events on the whole cohort, finding AUCs for this score of 67.0 (56.3-77.6) and 67.9 (57.8-78.1) at 3 and 5 years, respectively.

## Discussion

In this study, we investigated the relationship between some biomarkers of systemic inflammation and overall survival in HIV-infected patients with NHL for the first time. We found that some inflammatory-based scores including serum levels of CRP and albumin (GPS, mGPS, PI and PNI), but not those based on cellular components of the white cell count (NLR and PLR), had an independent prognostic value for risk of death. We also computed a mortality risk score including PNI and occurring of an AIDS event within six months from NHL diagnosis, which showed a rather fair predictive ability, with an AUC of 0.69 at 3 years of follow-up.

Various prognostic parameters have been used for NHL, so far. Among them, the IPI is probably the most commonly used and it has been revised recently [10,23,24]. New inflammatory indexes, based on routinely examined blood parameters, have been proposed, including NLR and GPS: NLR has been found useful in patients with diffuse large B-cell lymphoma at low risk of death [18], whereas an elevated GPS was associated with poorer survival in patients with extranodal natural killer/T-cell lymphoma [19] and in those with diffuse large cell lymphoma treated with R-CHOP [17].

In HIV-infected patients with NHL, however, also HIV-infection related factors, such as cART, concurrent infections and baseline immunosuppression, have been found to influence prognosis [4,7,25]. However, the studies carried out so far produced inconsistent results. A low CD4 cell count at NHL diagnosis found to be an independent risk factor for death in pre-cART and early cART eras in two large cohorts [3-5,26], whereas history of AIDS was associated with worse survival only in the pre-cART era [4]. A protective effect of cART has been described [27,28]. Other studies, however, found that only patient- and lymphoma-, but not HIV-related factors were predictive of poor outcome in the cART era [7-9]. For this reason, a composite score for HIV-infected patients with NHL has been recently developed which involves both HIV infection factors and the variables included in IPI [29]. Nevertheless, the IPI is not of common use for HIV patients, because of lack of some necessary data, and other inflammatory scores based on routine data, such as NLR and GPS, may be considered.

In our study, we have included all HIV-infected patients with NHL independently of histology, Ann Arbor stage or chemotherapy regimen. The 2-year cumulative risk of death from NHL diagnosis in our cohort was 45% [1], slightly lower than the 50-70% value reported in previous studies on HIV-infected subjects [4,5,26,30]. This is possibly due to the fact that previous studies included patients with NHL diagnosis between the late 1990s and early 2000s, when both cART and chemotherapy regimens were not so effective as they are todays.

In these subjects, we found that some indexes including serum CRP and albumin (GPS and mGPS, PNI and PI), as representative of systemic inflammation and nutrition status, were independent predictors of survival in HIV patients with NHL. Therefore, we developed a simple score which integrates clinical and laboratory variables for assessing risk of death in HIV-infected patients with NHL. The score includes one of the HIV-related variables, i.e. an AIDS defining event occurring within six months from NHL diagnosis, and one inflammatory score, the PNI, based on common blood parameters such as lymphocytes and albumin, which therefore can be easily applied to any patient with newly diagnosed NHL. We found that this score had a rather fair predictive ability with an AUC of 0.69 at 3 years of follow-up.

There are several strengths in our study, including a large sample size, multicenter national cohort and inclusion of patients receiving state of the art lymphoma and HIV care. This study has some limits, too. First, we could not analyze the prognostic factors according to type of NHL, due to lack of power of the study for subgroup analyses. Second, the derivation and validation samples used to compute and test the prognostic score were unbalanced (70% and 30%, respectively). In fact, because of the relatively small number of NHL cases, we choose to include more subjects (70%) in the derivation cohort to get better precision of the estimates. Anyway, we also computed a prognostic score including PNI and AIDS events within six months from NHL diagnosis on the whole cohort, finding an AUC for this score similar to those computed using validation and derivation cohorts.

## Conclusions

In summary, our study extends previous findings which were focused on solid cancers and shows that some markers of systemic inflammatory response are associated with poor outcome also in HIV-infected patients with NHL.

## Abbreviations

NHL: Non-hodgkin lymphoma
cART: Combined antiretroviral therapy
IPI: International prognostic index
LDH: Lactate dehydrogenase
CRP: C-reactive protein
GPS: Glasgow prognostic score
NLR: Neutrophil/lymphocyte ratio
PLR: Platelet/lymphocyte ratio
HBV: Hepatitis B virus; HCV: Hepatitis C virus
HCV: Viral hepatitis C
mGPS: Modified glasgow prognostic score
PI: Prognostic index
PNI: Prognostic nutritional index
HR: Hazards ratio
95% CI: 95% confidence interval
ROC: Receiver operating characteristic
AUC: Area under the receiver operating characteristic curve
SD: Standard deviation
Rm: Risk of mortality
